# A comparison of fast growing broiler chickens with a slower-growing breed type reared on Higher Welfare commercial farms

**DOI:** 10.1371/journal.pone.0259333

**Published:** 2021-11-04

**Authors:** Mary Baxter, Anne Richmond, Ursula Lavery, Niamh E. O’Connell

**Affiliations:** 1 Institute for Global Food Security, School of Biological Sciences, Queens University Belfast, Belfast, Northern Ireland; 2 Moy Park Ltd, Portadown, Craigavon, Northern Ireland; Tokat Gaziosmanpasa Universitesi, TURKEY

## Abstract

Slowing the growth of modern broiler chickens can have a positive effect on a number of welfare outcomes. However, relatively few studies have compared fast and slower growing broiler chickens reared under the same commercial conditions. The main aim of this study was to evaluate a slower growing breed and standard fast growing broilers on commercial farms. Ross 308 broilers and slower growing Hubbard Redbro broilers were housed on six farms for 17 production cycles. Production data were available for all cycles. Behaviour and environmental measures were taken over one cycle on each of two farms. The farms were visited during weeks 3–6 for both breeds and week 7 for Redbros. We found that breed had a significant effect on a number of measures, including gait score, latency to lie, feather cover, avoidance distances, perch use and play behaviour (p < 0.05). Gait scores were consistently lower among the Redbro flocks during weeks 4, 5, 6 and 7. Redbro broilers generally had longer latency to lie times, better feather cover, and were more reactive to approaching observers. They also showed higher levels of perch use and play. Despite these indications of improved locomotion and physical ability, we found little difference in their general behaviour. However, Redbro broilers did perform longer activity bouts in week 7 than Ross 308s in their final week. There was no effect of breed on dust levels, ammonia concentration or litter condition. Redbro broilers were slaughtered 5.5 days later than Ross 308 birds at a lower average weight (2.32 vs 2.52kg) and had lower mortality, fewer culls and fewer carcasses downgraded at the abattoir. Our results suggest that the slower growing strain was healthier throughout the cycle and more capable of displaying some natural behaviours.

## Introduction

The global poultry industry has expanded to provide over 72 billion chickens a year for meat [1]. This production level is possible largely due to the focussed selective breeding of broilers for performance traits. Modern broilers are characterised by their rapid growth rate, high feed efficiency and high meat yield. A slaughter weight of 2.5 kg can now be reached in 38 days compared to 63 days in the 1960s [2,3]. However, these intensive genetic traits have been associated with numerous welfare concerns, including low activity levels, leg disorders, contact dermatitis and metabolic issues (reviewed by [4,5]). High levels of chick mortality, late mortality, culling and carcass downgrades can also occur if these issues are severe, resulting in economic losses for the farmers and producers [5]. The broiler industry has come under sustained pressure from welfare organisations to mitigate the issues prevalent among flocks of fast-growing broilers. Recently, several major companies and retailers signed the European Chicken Commitment, in which they pledge to only source broilers reared to increased welfare standards by 2026 [6]. These standards include adopting breeds that demonstrate higher welfare outcomes compared to standard fast-growing broiler breeds. There has been a recent focus on the benefits of rearing slower growing strains of broiler chicken [7,8]. Although there is no common definition of a “slow growing broiler”, lower growth rates are commonly associated with improved leg health, an increase in activity levels and a reduction in contact dermatitis compared to faster growing broilers (e.g. [7–10]). There is also some evidence that slower growing broilers display more markers of positive welfare [7].

Slowing the growth of intensively reared broilers has been discussed for some time as a method of improving their health and welfare [11,12]. However, there are very few studies that evaluate slower growing broilers against their fast-growing counterparts at a commercial scale, and even fewer that incorporate welfare outcomes into the study. Therefore, the purpose of this study was to make a thorough comparison of a conventional fast-growing broiler breed and a slower growing breed that was being trialled by a leading poultry producer. Assessments of leg health were used alongside a variety of behavioural observations to evaluate the health and welfare of each breed on working farms. The effect of breed on litter quality, dust levels and ammonia concentration was also monitored to identify any difference in their environmental impact. The productivity of any slower growing breed remains an extremely important factor in their suitability for commercial use, and a detailed analysis of the abattoir data, mortality levels and cull levels for both breeds was performed.

## Materials and methods

### Animals and housing

All methods described in this paper were approved by the School of Biological Sciences (Queen’s University Belfast) Research Ethics Committee (reference number QUB-BE105AREC-17-001). This study was conducted between December 2019 and August 2020 on six Moy Park affiliated Higher Welfare farms in Northern Ireland over 17 production cycles (34 flocks). On each farm, two matched, metal framed houses were stocked simultaneously with either Ross 308 broiler chickens or slower growing Hubbard Redbro broiler chickens. Production data was acquired after slaughter and was available for all 34 flocks. Behavioural observations and on-farm assessments were made on two farms for one production cycle each, in July and August 2020.

For all farms, chicks were mixed sex with an approximate 50:50 ratio of male:female. There were slight variations in house size (from 73 m by 18 m to 85 m by 20 m) and flock sizes (from 21 500 to 28 000 broilers) between farms. However, stocking densities were maintained at ≤ 30 kg/m^2^ for all farms. For the two farms used for on-farm assessments, the houses on Farm 1 were 73 m by 19 m and on Farm 2 were 85 m by 20 m. This provided a total usable floor space of 1 324 m^2^ and 1 695 m^2^, respectively. Farm 1 was stocked with 21 500 broilers in each house. Farm 2 was stocked with 26 541 Redbro broilers and 27 200 Ross 308 broilers. Houses were initially bedded with woodshavings on Farm 1 and a straw pellet wood crumb mix on Farm 2, with both farms spreading short-cut straw to maintain litter condition throughout the cycle. As was standard practice for these farms, all houses were thinned (partial depopulation) towards the end of the production cycle, approximately a week before the remaining broilers were cleared at the final slaughter weight. All farms had houses fitted with windows that provided natural light between 09 00 h and 17 00 h. Artificial lighting was also provided in accordance with EU regulations (Council Directive 2007/43/EC), with the dark period gradually increasing to at least 6 h at day 7 until three days before slaughter. At three days before thinning, the dark period was reduced to 3 h and then reduced by 1 h per day until a dark period of 1 h was reached the day before thinning. That 1 h of darkness was then maintained until clearing. All houses were equipped with platform perches and short-cut plastic wrapped bales as environmental enrichment, which was standard for these Higher Welfare farms. Seven metal framed platform perches with white plastic gridding were available in each house (260 cm by 60 cm), with three on one side and four on the other. The perches were suspended from the ceiling and connected to winches, which allowed them to be raised at the farmer’s discretion to a height they considered them usable for the birds. This varied, as the slower growing Redbros were typically able to jump higher than the Ross 308s, however they tended to be around 20cm– 30cm from the floor at their highest. All houses were supplied with 40 straw bales at the beginning of the cycle, which were distributed by the farmer and gradually cut open to allow birds to scratch out and peck at the straw. All farms provided ad lib access to feed and water. Both breeds received the same feed; a commercially developed diet based on Aviagen specifications for Ross broilers [13]. Temperature and humidity were maintained automatically according to producer guidelines.

### Measurements

The two farms used for on-farm assessments were visited once a week in weeks 3, 4, 5, 6 and 7 (week 7 for Redbro broilers only). The same observer performed all behavioural tests and analysed all footage. It was not possible to blind the observer to breed, due to the physical differences between Redbro and Ross 308 broilers. However, detailed scoring systems and a broad range of quantifiable responses (including time and number of birds engaged in an activity) were used to give a thorough assessment of each breed and minimise bias. All video footage was taken using Camileo X-Sports cameras (Toshiba, Surrey, UK) and GeeKam action cameras (Shenzhen Bodalong Technology Co., Ltd, Guangdong, CN) mounted on 1 metre high tripods (AmazonBasics, London, UK).

#### Leg health

Leg health was assessed using a combination of gait scoring and latency to lie. Once a week, a total of 40 broilers were gait scored in each house. A numbered Perspex grid was used to select two broilers from each of twenty randomly chosen (10 central and 10 edge) sections of the house, as in [14]. The observer approached the bird and encouraged them to move away, their walking ability was then scored using the Garner et al. [15] method, on a scale of 0 (no impairment) to 5 (unable to stand). Once two broilers had been assessed for gait score, there was a 1 minute settling period before a third broiler was assessed for latency to lie. An adapted latency to lie test, without a water bath, was used to minimise stress for birds [16]. A seated broiler closest to random grid coordinates was chosen and the bird was slowly approached until it stood. A stopwatch was used to record the time spent standing before the broiler returned to a seated position. The test was terminated and the maximum score of 120 given if the broiler made no attempt to sit after 2 minutes.

#### Feather quality

All broilers that were assessed for walking ability (n = 40 per house per week) were also assessed for feather cover and feather cleanliness. Feather cover was measured on a scale of 0 (feather cover is full and even over body and wings) to 2 (body is bare of feathers and wings are patchy of feathers [17]). To minimise disturbance, broilers were not picked up to assess their underside. However the cleanliness of their feet, back, wings and upper chest was assessed on a scale of 0 (feathers white, no caked dirt on legs and feet), 1 (either moderate soiling all over body or variable soiling with no more than half the body or legs having caked dirt and most feathers free) or 2 (most of body and feet caked with dirt adhering the feathers to each other) [18].

#### General behaviour

Broiler behaviour in unenriched areas of the houses was assessed using a combination of scan and focal sampling. Each week, four randomly selected areas of the house that contained no perches or straw bales were video recorded in each house for half an hour simultaneously, giving a total of 2 hours of footage per house per week. Broilers inside a 2 m^2^ area in front of the camera were scan sampled at 10, 15, 20, 25 and 30 minutes. The number of broilers inside the area was recorded, and they were categorised as either dustbathing, foraging, sitting inactive, sitting pecking, in locomotion (standing or walking), sitting preening, standing preening or other (Table 1). Further to this, of the birds in a seated position, the percentage sitting inactive and the percentage resting were calculated. Focal observations of activity bouts were also conducted to determine how long a broiler remained in activity after standing before returning to a seated position. The first 10 birds to stand after a 10 minute settling period were observed per video, per week, per house (broilers observed N = 710; data for one video were missing (N = 10)). The bird was observed and the time recorded from the time they stood to the time they returned to a seated position. If a bird left the frame then another bird was chosen, although this was not common.

**Table 1:** Ethogram used to record broiler chicken behaviour (Based on [19]).

**General behaviour**	
Foraging	Scratching and pecking at the ground (from a standing or walking position)
Sitting inactive	Sitting down without performing ground pecking or any other behaviours. The broilers eyes are open and the head is not tucked under a wing.
Sitting pecking	Ground pecking from a seated position
Locomotion	Walking (taking more than one pace in any direction) or standing with no other activity.
Sitting preening	The bird runs their beak through their feathers in a seated position
Standing preening	The bird runs their beak through their feathers in a standing position
Resting	The bird sits with its eyes closed, or with its head beneath one wing/ resting on the ground, or the bird lies on one side with or without its eyes closed.
Dustbathing	Broilers are lying and performing head rubbing, vertical wing-shakes, leg scratching, and/or raking the substrate closer to them with their beak. Broilers clearly covered in substrate and lying without clearly performing other behaviours are categorised as dustbathing because the end of a dustbathing bout is typically signified by a body-shake which removes excess substrate. Broilers preening while covered in substrate are classified as dustbathing. Broilers not covered in substrate and performing preening without any additional dustbathing behaviours are classified as preening.
Other	Any other behaviour, including eating and drinking.
**Play behaviour**	
Sparring	A bird simulates fighting behaviour with no obvious aggression or injurious contact. The following behaviours may begin a bout and occur during a bout: jumps with light kicking that make little or no contact with the receiver; stand-offs (threats) in which birds will face up to one another briefly, stepping close to one another and raising their necks to stand practically beak-to-beak (with or without a difference in head height); raising feathers around the neck, usually during a stand-off; stand-off with wing-flapping; stand-off with light pecks at the neck, head or beak of the receiving bird. These differ from aggressive actions in that they are not forceful, prolonged and they do not elicit strong avoidance from the receiver. It would be difficult to estimate a pecking order based on these behaviours. The bird that these behaviours are directed at may or may not respond, in some cases birds attempt a stand-off with a seated bird and are ignored. Birds usually end the short behaviour by sitting down or engaging in another activity.
Food-running	A bird picks up the straw and runs or moves away quickly, often running and making counter-intuitive direction changes towards conspecifics. There are conspicuous peeping noises that typically accompany this behaviour. Conspecifics chase the lead bird, and the object may move between several birds.
Frolicking	Spontaneous and rapid running and/or jumping and wing-flapping with no obvious intention, often with rapid direction changes. Running without wing-flapping is not classified as frolicking. A frolicking bout ends when the bird sits down or resumes another activity. Birds displaying frolicking directly leading to sparring within the frame are categorised as sparring if there was no break between the behaviours.

#### Perch use

Perch use was assessed using three measures: the number of birds on the perch (perch occupancy), the number and success of any perching attempts, and the time spent perching. All measures were taken using video footage of four randomly chosen perches, from the available seven perches. The perches were recorded for half an hour each, between 12 00 h and 17 00 h, giving a total of 2 hours of video footage per house, per week. For perch occupancy, following a 10 minute settling period the number of birds on the perch was counted during a scan sample every 5 minutes (at 10, 15, 20, 25 and 30 minutes) and an average given for each observation period. After each 5 min scan, the perches were observed for a two minute focal period and all attempts to perch recorded. These attempts were classified as either successful (the broiler alighted on the perch) or failed, and averaged over the observation period. Time spent perching was recorded for the first five broilers to successfully perch following the 10 minute settling period. A perching bout began when the bird landed on the perch and ended when they jumped off the perch. If a broiler fell or jumped off the perch less than 3 seconds after jumping on then another bird was chosen. However, for 48 of the 242 perching bouts observed, the focal broiler was still on the perch at the end of the observation period (when the bird was then disturbed off the perch by the returning observer). The time these birds had been perching for varied (e.g. some had been perching for 20 mins and some for 2 mins). Including the perch times of broilers that had been disturbed soon after they jumped onto the perch may have led to an underestimating of natural perching bout lengths. Therefore, any broiler that had been perching for less than 8 minutes was disregarded. A limit of 8 minutes was chosen as this was the average perch time for broilers that voluntarily left the perch during the observation period (n = 242, M = 466.22 s). In several videos, no or very few Ross 308 broilers jumped onto the perches. Our analysis is therefore assessing the difference in perch time of the subsample of broilers that chose to perch during the observation (Redbro N = 192, Ross 308 = 90).

#### Avoidance behaviour

Fearfulness was measured once per week per house, using avoidance testing as in [19]. Ten broilers were chosen from 10 randomly selected sections of the house using a Perspex grid with random coordinates. Each broiler was approached from a distance of ~ 5 m, at a speed of approximately 1 step/sec. At the point that the broiler withdrew by lifting their second foot, a line in the litter was drawn at the toe of the observer’s boot and the observer placed the tape measure between the last place the broiler had been and the line in the litter. The distance between the observer and the broiler’s point of withdrawal was measured in cm.

#### Response to novel object

A novel object test, based on [16], was performed in two randomly selected areas of each house (one central and one edge) per week. The observer placed the object in the centre of the section and walked away to a distance of ~ 3 m. The latency for a bird to peck at the object was recorded with a stopwatch. The number of birds that pecked the novel object over a 300s period and the number within 50 cm of the object at the end of this period was also recorded. If no bird approached the novel object within 5 minutes then a maximum latency of 300 s was recorded and the test was terminated. The novel objects used were as follows for both farms: week 3 = a multi-coloured children’s ball, week 4 = a child’s lime green plastic chair, week 5 = a 2 L bottle of orange liquid, week 6 = a small orange traffic cone, week 7 = an upturned small blue bucket.

#### Play behaviour

Play behaviour was stimulated by a walk through, to assess frolicking and sparring, and by throwing sections of paper straw to stimulate food-running. Walk-throughs were performed as in [19]. In four randomly chosen locations, a camera was set up between a feeder and drinker line, facing the back of the house. The camera view took in at least 2 metres of floor in front of it. The observer walked 5 metres in front of the tripod and back towards it, clearing a space in front of the camera. The observer left the house and continued to record for 10 minutes. All occurrences of sparring and frolicking within a 2 metre space in front of the camera were recorded in the 5 minutes after the birds were displaced (Table 1). Food-running was assessed in 10 randomly selected areas, balanced for edge and central sections (N = 180; Table 1). The observer stood still for a 2 minute settling period and then threw a 7 cm section of striped red and white paper straw (The British Straw Company, Cheshire, UK) approximately 1 m ahead of them. The observer remained still and recorded the presence or absence of food-running over a 1 minute observation period. Pecking at the straw was not classified as food-running (Table 1).

#### Environmental measures

Litter quality was assessed once per week using a transect method adapted from the Welfare Quality Protocol [18] to give an extensive overview of the house litter condition. The observer started at the front of the right side of the house and walked towards the back of the house between a feeder and drinker line, stopping every 12 steps to assess the litter between and including the neighbouring feeder and drinker lines. The observer repeated this process returning down the central line of the house, and then back up the left side of the house, recording a total of 30 locations. Litter was scored at each stop on a scale of 0 (completely dry and flaky) to 4 (sticks to boots once the cap is broken [18]). Dust levels (Split2 Particulate Monitor, SKC Ltd, Dorset, UK) were recorded in one central location in the house before any other observations began once per week. Ammonia levels (BW Gasalert Extreme Gas Detector, Safety Gear Store, Staffordshire, UK) were measured in the same four locations around the house each week.

#### Production data

Production data was taken from slaughter records provided at the end of each production cycle (N = 17, a total of 762 079 broilers). Information on three types of contact dermatitis were taken at the abattoir: footpad dermatitis, breast burn and hock burn. All carcases were tested automatically for hock burn and breast burn using computer visualisation. All lesions > 3mm were recorded. Footpad dermatitis was recorded by hand, with a random sample of 100 carcases per house monitored and feet scored by abattoir staff. A scoring system of 0–2 was used, where ‘0’ represents either no pododermatitis or very superficial lesions, ‘1’ represents mild pododermatitis on either foot with discolouration of the footpad and superficial lesions, and ‘2’ was recorded when there was severe pododermatitis on either foot with ulcers, signs of haemorrhage and/or swollen footpads. Breast burn was rare and is not reported here.

### Statistical analysis

All data were analysed using SPSS v26. A linear mixed model was used to assess the effects of Breed, Week and their interaction, with Farm as a random factor, for weeks 3–6 of the following variables, i) latency to lie (N = 320), ii) percentage of general behaviours observed during scans (N = 63), iii) percentage of resting among seated broilers (N = 63), iv) length of activity bout (N = 315), v) perch occupancy (N = 63), vi) total perching attempts (N = 63), vii) percentage of successful perching attempts (N = 63), viii) time spent perching (N = 282), ix) total play bouts (N = 63), x) avoidance distance (N = 160). The vast majority of preening was performed from a seated position (seated preening = 91%, standing preening = 9%), and preening categories were combined to a single “preening” measure. Stocking density, as birds per m^2^, within the scan sampling area was initially included in the model but was not significant for any behaviours and was removed. Where outcomes did not satisfy normality assumptions, they were log or square root transformed to improve the normality of residuals prior to analysis. If normality could not be improved with transformation then non-parametric methods were used. To directly compare the behaviour of each breed at slaughter weight, a separate comparison of Ross 308s in week 6 and Redbro broilers in week 7 was made using either the same model as applied to the week 3–6 data, independent samples t-tests or Mann Whitney U tests. Gait score (N = 640) and feather cover (N = 640) results were considered ordinal and were analysed using a Mann Whitney U test to compare the distribution of scores in Ross 308s and Redbro broilers within week for weeks 3–6 and then for a comparison between week 6 Ross 308 and week 7 Redbros. Feather cleanliness scores were low throughout (indicating clean feathers) and were not statistically analysed. Dust level (N = 16) comparison by breed was made using a Mann Whitney U test. Weeks 3–6 of ammonia levels were log transformed prior to analysis and analysed with a linear model with breed and week as fixed effects. Slaughter data were primarily analysed with a mixed model, with Breed as a fixed factor and Farm and Cycle as random factors. All percentage data were converted into proportions and logit transformed prior to analysis to satisfy the assumptions of linear models. Food-running was successfully stimulated in the majority of tests (N = 160), and as such statistical analysis was not deemed necessary. Presence or absence of food-running was converted into the percentage of “successful” tests, ie. tests in which food-running was observed, over the 10 randomly selected areas per week (N = 18) and descriptives are presented. Kruskall-wallis and Mann Whitney U tests were used to determine the effect of breed and week on i) time to peck at novel object (N = 32), ii) no of birds that pecked at the novel object (N = 32) and iii) the number of birds within 50 cm of the novel object after 5 minutes (N = 32). Bonferroni correction for multiple comparisons was applied to i) type of cull data, ii) behaviour scan data, iii) downgrade data, and iv) mortality and cull data analysed by day. Descriptive data of means (M) and standard deviations (±) presented throughout.

## Results

### Leg health

There was no significant difference between the gait scores of Ross 308 broilers and Redbro broilers in week 3 (p > 0.05). However, in weeks 4, 5 and 6 there was a significantly higher proportion of Redbro broilers with lower gait scores compared to Ross 308s (Table 2). In their final weeks, 7 week old Redbro broilers also had a significantly higher proportion of low gait scores compared to 6 week old Ross 308s (week 6, Ross 308 mean rank = 87.26, week 7, Redbro mean rank = 73.74, N = 160, p = 0.043). For interpretation, mean gait scores were as follows: week 3 Ross = 0.26 and Redbro = 0.14, week 4 Ross = 0.46 and Redbro = 0.16, week 5 Ross = 0.88 and Redbro = 0.43, week 6 Ross = 1.16 and Redbro = 0.57, week 7 Redbro = 0.94.

**Table 2:** Distribution of the frequencies of broiler gait score (%; N = 640). Data were considered ordinal and were analysed using Kruskall-Wallis tests. Mean rank and the test statistic (U) presented, with a p value < 0.05 indicating a significant difference in gait score between the two breeds at that age. A higher gait score (GS) indicates a worse walking ability.

	**Week 3**									
**Breed**	GS0	GS1	GS2	GS3	GS4	GS5	N	Mean rank	U	p value
Redbro	86	14	0	0	0	0	80	76.36	-	> 0.05
Ross 308	76	21	3	0	0	0	80	84.64		
	**Week 4**									
	GS0	GS1	GS2	GS3	GS4	GS5				
Redbro	84	16	0	0	0	0	80	68.92	4126.5	< 0.001
Ross 308	55	44	1	0	0	0	80	92.08		
	**Week 5**									
	GS0	GS1	GS2	GS3	GS4	GS5				
Redbro	61	35	4	0	0	0	80	68.98	4122.0	0.001
Ross 308	38	44	15	1	3	0	80	92.03		
	**Week 6**									
	GS0	GS1	GS2	GS3	GS4	GS5				
Redbro	58	28	15	0	0	0	80	63.99	4521.0	< 0.001
Ross 308	16	55	26	1	1	0	80	97.01		
	**Week 7**									
	GS0	GS1	GS2	GS3	GS4	GS5				
Redbro	30	50	18	1	1	0	80			0.043^1^
	**Overall**									
	GS0	GS1	GS2	GS3	GS4	GS5				
Redbro	64	29	7	0	0	0	400	331.64	75543.5	< 0.001
Ross 308	46	41	11	1	1	0	320	396.57		

^1^p value for a slaughter weight comparison between week 7 Redbro and week 6 Ross 308 broilers. Mean ranks and test statistics in the text.

There was a significant effect of breed on latency to lie, with Redbro broilers (31.67 ± 30.06 s) taking longer to return to a seated position than Ross 308 broilers (22.86 ± 22.45 s; N = 320, F(1,1) = 762.49, p = 0.023). There was no significant effect of age on average latency to lie (p > 0.2; week 3 = 36.08 s, week 4 = 22.49 s, week 5 = 29.58 s, week 6 = 20.89 s). There was also no significant difference between 6 week old Ross 308 broilers (28.17 s) and 7 week old Redbro broilers (20.94 s; N = 80; p > 0.3). There was no significant interaction between breed and week.

### Feather quality

There was no difference in feather cover between Redbro broilers and Ross 308 broilers in week 3. However, in the remaining weeks there were a significantly higher proportion of Redbro broilers with lower feather cover scores compared to Ross 308 broilers, indicating a better feather cover (Table 3). Slaughter weight comparison showed that Redbro broilers still had significantly higher proportion of low feather cover scores in week 7 compared to Ross 308 broilers in week 6 (Ross 308 mean rank = 85.00, Redbro mean rank = 76.00, N = 160, U = 3560, p = 0.021). For interpretation, mean feather cover scores were as follows: week 3 Ross 308 = 0.26 and Redbro = 0.26, week 4 Ross 308 = 0.53 and Redbro = 0.45, week 5 Ross 308 = 0.36 and Redbro = 0.15, week 6 Ross 308 = 0.08 and Redbro = 0.01, week 7 Redbro = 0.03. The initial increase in score after week 3 is likely to be due to feather down falling out and being replaced with new feathers, leaving cover patchier. Both breeds were generally clean on these two farms. There was occasional discolouration of feathers but rarely did chosen birds have caked dirt or excessive soiling that would grade higher than a 0. Throughout the study, only 5 Redbro broilers were scored higher than 0, and only 2 Ross 308s; all 7 birds were scored above 0 in the final two weeks of their production cycles.

**Table 3:** Distribution of the frequencies of feather cover scores (%; N = 640). Data were considered ordinal and were analysed using Kruskall-Wallis tests. Mean rank and the test statistic (U) presented, with a p value < 0.05 indicating a significant difference in feather cover between the two breeds at that age A higher feather cover (FS) score indicates a worse level of feather cover.

	**Week 3**									
**Breed**	FS0	FS1	FS2	FS3	FS4	FS5	N	Mean rank	U	p value
Redbro	48	53	0	0	0	0	80	80.50	-	> 0.9
Ross 308	48	53	0	0	0	0	80	80.50		
	**Week 4**									
	FS0	FS1	FS2	FS3	FS4	FS5				
Redbro	10	90	0	0	0	0	80	74.40	3688	0.003
Ross 308	4	86	10	0	0	0	80	86.60		
	**Week 5**									
	FS0	FS1	FS2	FS3	FS4	FS5				
Redbro	70	30	0	0	0	0	80	66.65	4308	< 0.001
Ross 308	4	86	10	0	0	0	80	94.35		
	**Week 6**									
	FS0	FS1	FS2	FS3	FS4	FS5				
Redbro	98	3	0	0	0	0	80	75.00	3640	< 0.001
Ross 308	84	16	0	0	0	0	80	86.00		
	**Week 7**									
	FS0	FS1	FS2	FS3	FS4	FS5				
Redbro	95	5	0	0	0	0	80			0.021^1^
	**Overall**									
	GS0	GS1	GS2	GS3	GS4	GS5				
Redbro	64	36	0	0	0	0	400	324.54	78384	< 0.001
Ross 308	43	51	5	0	0	0	320	405.45		

^1^p value for a slaughter weight comparison between week 7 Redbro and week 6 Ross 308 broilers. Mean ranks and test statistics in results section.

### General behaviour

**Fig 1:**
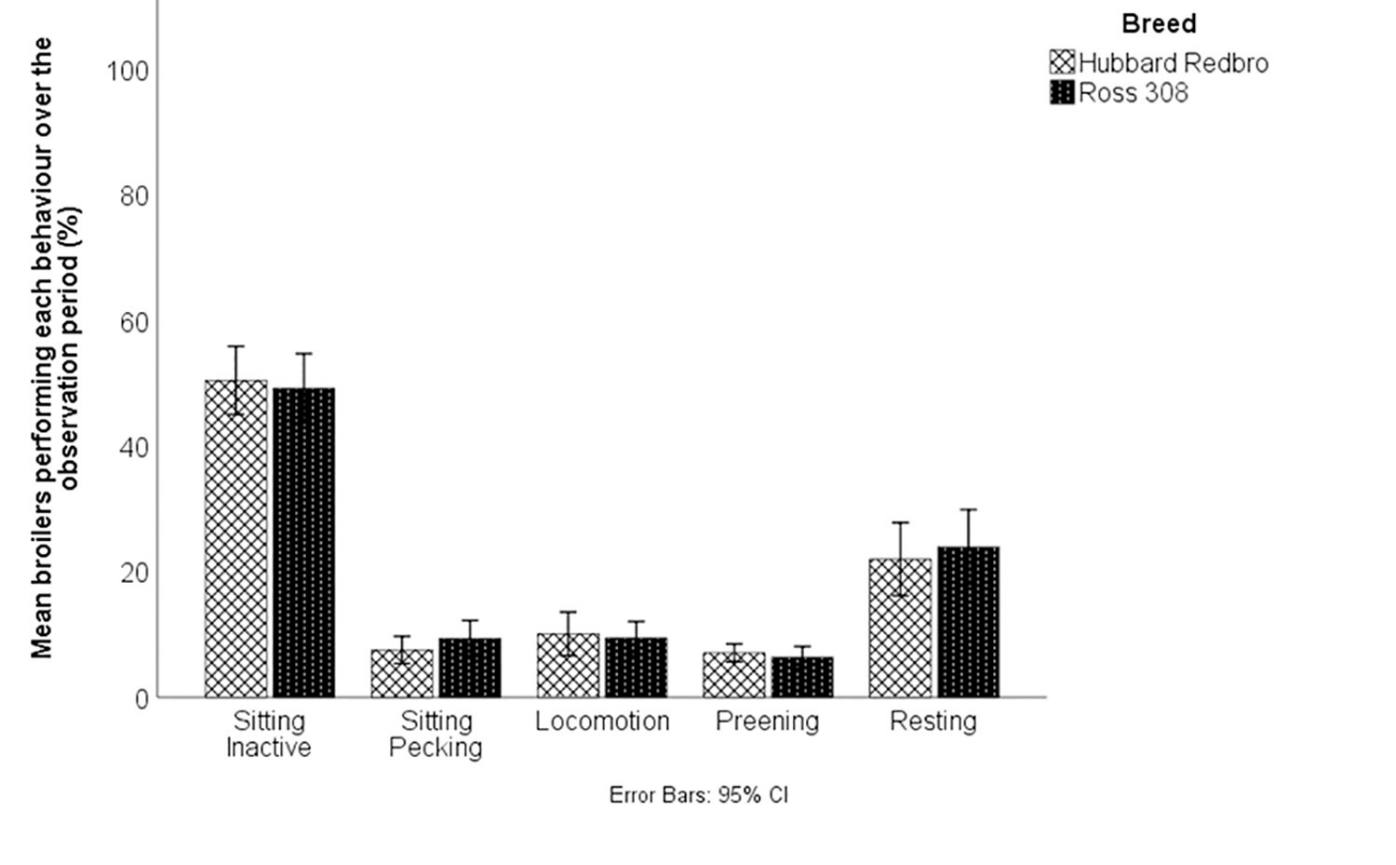
The overall behaviours observed in Redbro and Ross 308 broiler chickens. Data represent the mean percentage of behaviours over observation periods in week 3–6 of the production cycle. Foraging and Other were infrequently seen and were excluded from analysis.

**Fig 2:**
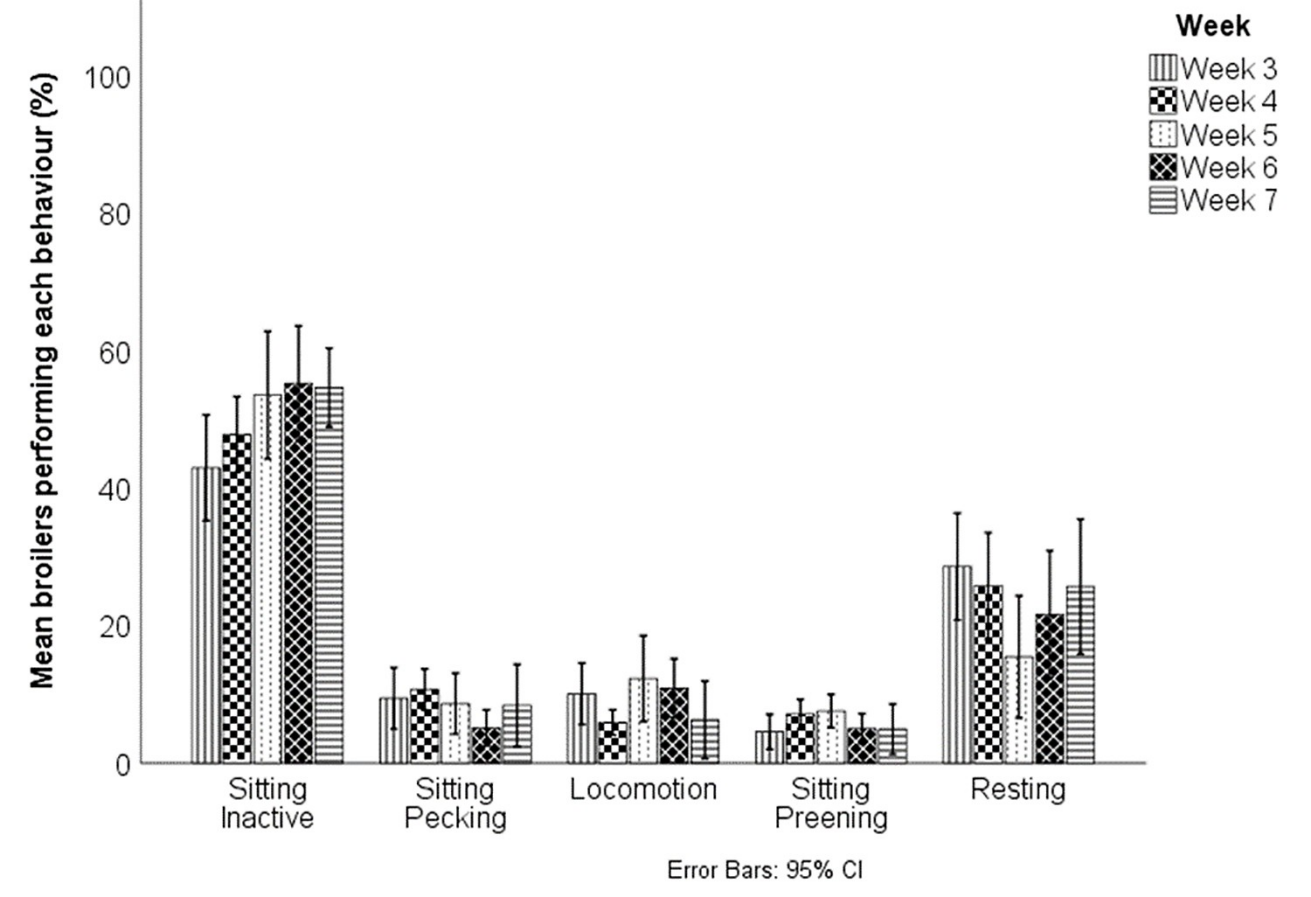
The overall behaviours observed in Redbro and Ross 308 broiler chickens, by week. Mean percentage of behaviours observed in Redbro and Ross 308 broiler chickens, by week. Foraging and Other were infrequently seen and excluded from analysis. Week 3–6 of the production cycle consists of both Redbro and Ross 308 data, and week 7 consisted of only Redbro behaviour data.

There was no significant effect of breed or week on the average proportion of broilers sitting inactive, sitting pecking, in locomotion, preening or resting during scan sampling (Figs 1 and 2). There were too few incidences of dustbathing and foraging for statistical analysis; descriptive results are presented. A total of 33 observations of foraging were observed in all scan observations (N = 144), 19 by Redbro broilers and 14 by Ross 308 broilers. There were 34 incidences of dustbathing recorded, 11 in the Ross broilers and 23 in the Redbros, with 13 recorded in one video of Redbros with a group of birds dustbathing together. There was no significant effect of breed on any behaviours between week 6 Ross 308 broilers and week 7 Redbro broilers (p > 0.1 for all). There was no effect of breed on the proportion of seated broilers resting, with a similarly low percentage of broilers resting (Redbro resting = 29.61%; Ross 308 resting = 32.06%). Week did have a significant effect on the proportion of seated birds that were resting (F(3,54) = 3.744, p = 0.016; week 3 M = 39.81 ± 17.08%, week 4 = 33.81 ± 16.43%, week 5 21.93 ± 21.54%, week 6 = 27.50 ± 22.19%), with post-hoc testing showing a significant difference between week 3 and week 5 (p < 0.05). There was no significant difference in the proportion of seated birds resting between Ross 308s in week 6 and Redbro broilers in week 7 (P > 0.05). The average length of time before broilers returned to a seated position after starting an activity bout was 30.86 s for Redbro broilers and 31.53 s for Ross 308 broilers. There was no significant effect of breed (p > 0.8) or week (p > 0.4) on the length of activity bouts in weeks 3–6. However there was a significant difference between Ross 308 broilers in week 6 and Redbro broilers in week 7 (F(1,157) = 5.107, p = 0.025), with Redbro broilers (46.98 ± 39.30 s) performing significantly longer bouts compared to Ross 308s (35.69 ± 36.23 s).

### Perch use

**Fig 3:**
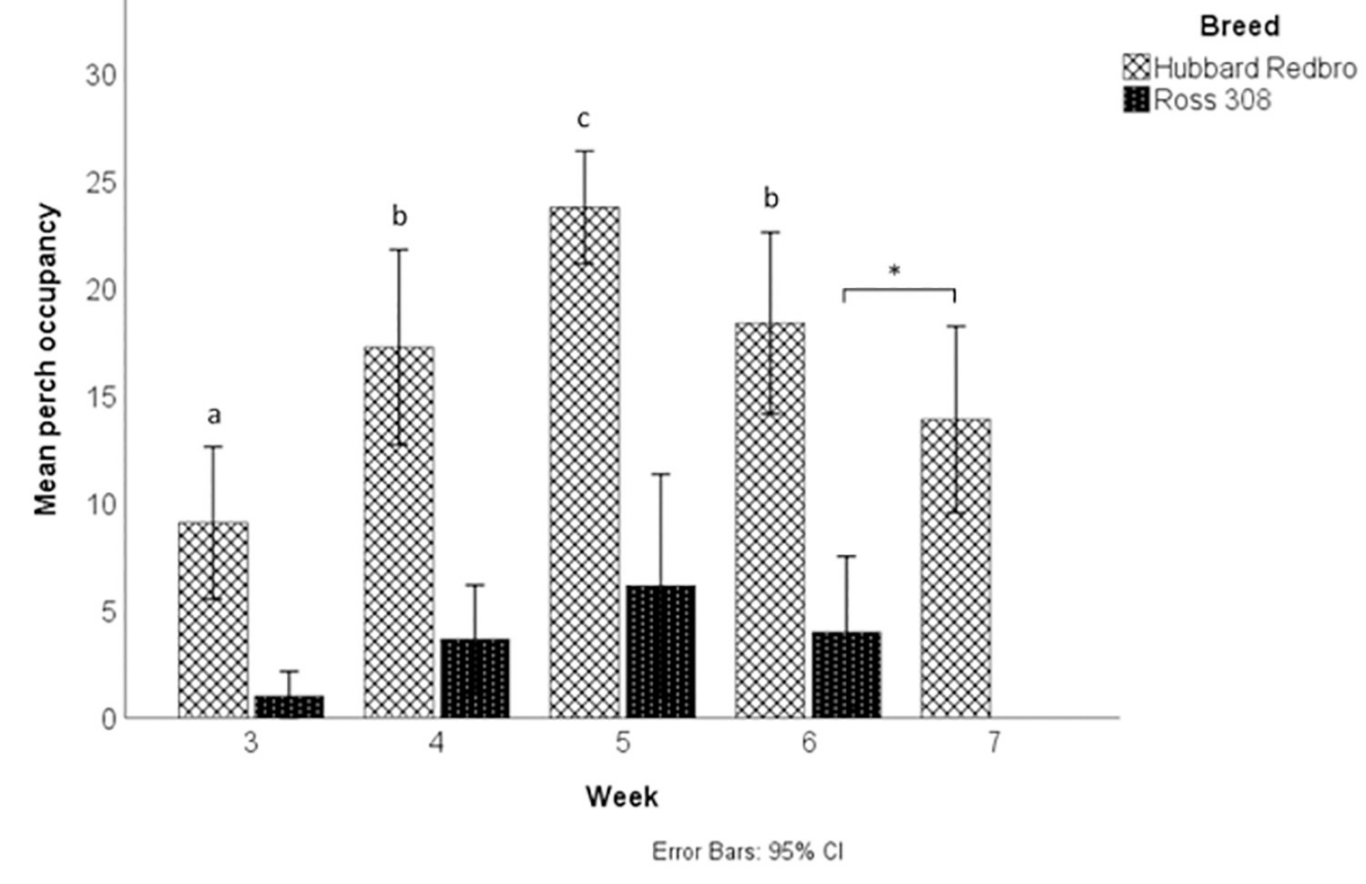
Perch occupancy results for Redbro and Ross 308 broiler chickens. Mean number of broilers on top of the platform perch, by week and breed. Different letters denote significant difference (p < 0.05) between weeks for Redbro broilers, following simple effects post-hoc analysis. * denotes a significance between 7 week old Redbro broilers and 6 week old Ross 308 broilers.

There was a significant interaction between breed and week for the average number of broilers perching during observations in week 3–6 (F(3, 54) = 3.686, p = 0.017; Fig 3). An analysis of simple effects showed that age had a significant effect on perch occupancy for the Redbro broilers (F(3,53) = 16.303, p < 0.001) but not for Ross 308 broilers (p > 0.1). For Redbro broilers, perch occupancy increased significantly until week 5 and then decreased in week 6 (Fig 3). There was a numerically large difference in perch occupancy between the two breeds, with an average of 16.43 Redbro broilers perching during observations compared to 3.70 Ross 308 broilers. There was also a significant difference in perch occupancy between Redbro broilers in week 7 and Ross broilers in week 6, with more Redbro observed using the perches at slaughter weight compared to the Ross 308s (U = 4.00, p = 0.002, Redbro mean rank = 12.00, Ross 308 mean rank = 5.00; Fig 3).

There was a significant effect of breed (F(1,54) = 48.33, p < 0.001) and week (F(3,54) = 2.97, p = 0.040) on the total number of perching attempts. The average number of perching attempts recorded per observation period was significantly higher for Redbro broilers (M = 2.92 ± 1.52) compared to Ross 308s (M = 0.91 ± 1.11). Total perching attempts tended to increase until week 5 and then declined in week 6 (week 3 = 1.77 ± 1.68, week 4 = 2.15 ± 1.72, week 5 = 2.40 ±1.99, week 6 = 1.28 ± 1.05). Post-hoc analysis revealed a significant difference between week 5 and week 6 (p = 0.046). There was also a significant difference between Redbro broilers in week 7 and Ross 308 broilers in week 6 at their respective final weights (U = 7.50, p = 0.007), with a higher number of Redbros observed making perching attempts compared to the Ross 308s (Redbro = 2.45 ± 1.39, Ross = 0.68 ± 0.89). Out of all attempts recorded, Redbro broilers were successful in 93% of cases and Ross 308 broilers were successful in 75% of cases. Analysis of the average percentage of successful perch attempts made per observation period showed a significant breed*week interaction (F(3,43) = 4.12, p = 0.012). Simple effects analysis revealed that a significantly higher percentage of perching attempts were successful in Redbros compared to Ross 308s in week 3 (Redbro successful attempts M = 95.11 ± 8.53%, Ross 308 = 47.50 ± 14.50%) and week 5 (Redbro = 94.83 ± 6.42%, Ross 308 = 67.89 ± 31.76%), but not in week 4 (Redbro = 90.80%, Ross 308 = 85.86%) or week 6 (Redbro = 86.25%, Ross 308 = 87.73%). There was no significant difference in the level of perching success between Ross 308 broilers in week 6 and Redbro broilers (96.88%) in week 7 (p > 0.05).

There was no significant effect of breed (p > 0.1) on time spent perching in weeks 3–6, with Ross 308 broilers spending an average of 636.80 seconds (10.6 minutes) and Redbro broilers an average of 487.72 s (8.1 minutes) on the perch. There was a significant effect of week (F(3,229) = 7.99, p < 0.001). Time spent perching increased significantly after week 3, with post hoc tests showing significant differences between week 3 and weeks 5 and 6 (p < 0.05; week 3 M = 330.12 ± 422.63 s, week 4 = 579.20 ± 587.71 s, week 5 = 721.03 ± 550.0 s, week 6 = 693.48 ± 604.05 s). There was no significant difference between Ross 308 broilers in week 6 (699.00 ± 643.83 s) and Redbro broilers in week 7 (598.88 ± 590.55 s; p > 0.05).

### Avoidance behaviour

There was a significant effect of breed (F(1,151) = 5.08, p = 0.026) on average withdrawal distance. Redbro broilers recording an average of 218.50 cm and Ross 308 broilers an average of 193.67 cm, which means that an observer could get closer to a Ross 308 broiler before they withdrew compared to a Redbro broiler. Withdrawal distances were also significantly affected by week, with avoidance increasing in week 4 and gradually reducing until week 6 (F(3,151) = 3.16, p = 0.027; week 3 = 217.40 cm, week 4 = 227.83 cm, week 5 = 190.75 cm, week 6 188.38 cm). No significant difference between weeks was detected in post-hoc analysis, although there was a trend for a difference between week 4 and week 6 (p = 0.074). There was no significant interaction between breed and week (p > 0.6). There was also no significant difference between Redbro in week 7 (Redbro = 204.95 cm) and Ross broilers in week 6 (Ross 308 = 177.90 cm; p > 0.05).

### Response to novel object

**Fig 4:**
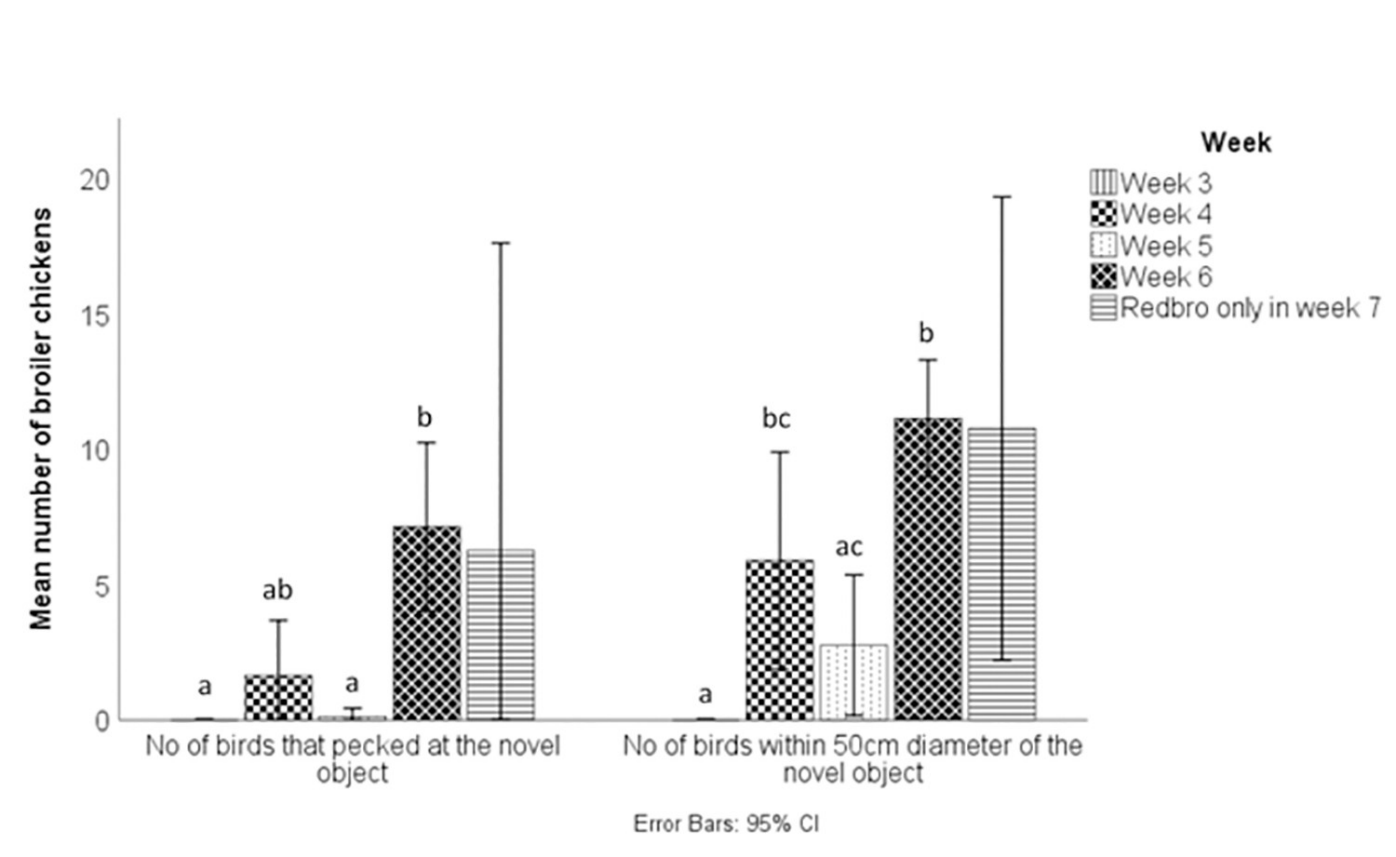
The response of broiler chickens to novel objects over the production cycle. The number of birds that pecked at the novel object during the test, and the number of birds within a 50 cm diameter of the novel object are presented. Different letters denote significant differences (p < 0.05) between the weeks within each test, for weeks 3–6 of the production cycle.

There was no significant effect of breed on the average time taken to peck a novel object (p > 0.6; Redbro M = 223.57 s, Ross 308 = 244.55 s), the number of birds that pecked at the novel object (p > 0.8; Redbro M = 2.38, Ross 308 = 2.06) or the number of broilers within 50 cm of the novel object after 5 minutes (p > 0.6; Redbro M = 5.44, Ross 308 = 4.44). There was a week effect on all three variables tested. For the time taken to peck (H(3) = 21.37, p < 0.001), older birds took less time to approach the novel object (week 3 mean rank = 23.00, week 4 = 15.88, week 5 = 21.50, week 6 = 5.63; Fig 4). Post hoc testing revealed a significant difference between week 6 and weeks 3 and 5 (p < 0.05). The number of broilers that pecked at the novel object was also affected by week (H(3) = 22.01, p < 0.001; Fig 4), with a higher number of birds pecking at the novel object in weeks 4 and 6. There was a significant post hoc difference between week 6 and weeks 3 and 5 (p < 0.05; week 3 mean rank = 10.00, week 4 = 17.06, week 5 = 11.38, week 6 = 27.56; Fig 4). The number of broilers recorded within a 50 cm distance of the novel object also increased in older birds (H(3) = 21.89, p < 0.001; week 3 mean rank = 6.00, week 4 = 19.06, week 5 = 14.06, week 6 = 26.88; Fig 4). Post-hoc tests revealed a significant difference between week 3 and weeks 4 and 6, and between weeks 5 and 6 (p < 0.05). There was no significant difference between 6 week old Ross 308 broilers and 7 week old Redbro broilers (p > 0.05).

### Play behaviour

There was a significant effect of breed (F(1,54) = 4.83, p = 0.032) and week (F(3,54) = 6.20, p = 0.001) on the total number of play bouts (frolicking and sparring) recorded in weeks 3–6. Redbro broilers performed an average of 40 (± 29.26) bouts of play compared to 28.16 (± 19.05) in Ross 308 broilers. Low play levels in week 3 increased in weeks 4 and 5 before reducing again in week 6 (week 3 = 29.40 ± 31.59, week 4 = 48.38 ± 25.42, week 5 = 38.81 ± 20.36, week 6 = 19.81 ± 13.02). There were significant pairwise comparisons between week 3 and weeks 4 and 5, and between week 4 and week 6 (p < 0.05). There was no significant effect of breed on the number of play bouts recorded in 6 week old Ross 308s (19.63 ± 16.85) compared to 7 week old Redbro broilers (26.83 ± 18.89; p > 0.5).

Food-running was observed in 139 out of a total of 180 tests. There was little numerical difference between the breeds, with an average 74% successful tests in Ross 308s and 75% in Redbro broilers. Prevalence of food-running did appear to be affected by age, with low levels of food-running seen in week 3 and similarly high levels seen in weeks 4, 5 and 6 (week 3 = 10% ± 8.16, week 4 = 90% ± 14.14, week 5 = 100% ± 0, week 6 = 98% ± 5.0). There was also very little difference between Ross 308 broilers in week 6 (95% successful tests) and Redbro broilers in week 7 (100% successful tests).

### Environmental measures

Overall, the mean litter score in the Redbro housing was 0.33 ± 0.52 and for Ross 308s was 0.44 ± 0.57. There was a significant difference in litter condition between Ross 308 broilers and Redbro broilers in week 4 only (Ross 308 mean rank = 70.57, Redbro mean rank = 50.43, N = 120, p < 0.001), with lower litter scores in the Redbro housing (Table 4). There was no difference in the houses at slaughter weight, with Redbro broilers in week 7 and Ross broilers in week 6 recording a similar litter condition. For interpretation, mean litter scores were as follows; week 3 Ross 308 = 0.12 and Redbro = 0.17, week 4 Ross 308 = 0.48 and Redbro = 0.13, week 5 Ross 308 = 0.68 and Redbro = 0.47, week 6 Ross 308 = 0.47 and Redbro = 0.50, week 7 Redbro = 0.52. There was no significant difference in dust levels between Redbro broilers and Ross 308 broilers overall (Redbro median dust level = 2.43 mg/m^3^, Ross 308 median dust level = 2.73 mg/m^3^, N = 16, p > 0.2). Ammonia levels were similarly unaffected by breed (p > 0.05), however there was a significant effect of week (F(1,71) = 8.68, p < 0.001). Ammonia levels increased across the cycle (week 3 M = 5.69 ± 6.47 ppm, week 4 = 11.25 ± 10.95 ppm, week 5 = 11.44 ± 8.77 ppm, week 6 = 20.94 ± 7.45 ppm). Post-hoc tests revealed a significant difference between week 3 and weeks 5 and 6, and between week 4 and week 6. There was no difference at slaughter weight, with Ross 308 broilers in week 6 and Redbro broilers in week 7 showing similar levels of ammonia.

**Table 4:** Distribution of the frequencies of litter scores (%; N = 540). Data were considered ordinal and were analysed using Kruskall-Wallis tests. Mean rank and the test statistic (U) presented, with a p value < 0.05 indicating a significant difference in litter condition between the two breeds at that age. A higher litter score (LS) indicates a worse litter condition.

	**Week 3**								
**Breed**	LS0	LS1	LS2	LS3	LS4	N	Mean rank	U	p value
Redbro	98	2	0	0	0	80	58.48	-	0.093
Ross 308	92	5	3	0	0	80	62.52		
	**Week 4**								
	LS0	LS1	LS2	LS3	LS4				
Redbro	87	13	0	0	0	80	50.43	2404	< 0.001
Ross 308	53	45	0	0	0	80	70.57		
	**Week 5**								
	LS0	LS1	LS2	LS3	LS4				
Redbro	53	47	0	0	0	80	55.60	-	0.082
Ross 308	42	48	10	0	0	80	65.40		
	**Week 6**								
	LS0	LS1	LS2	LS3	LS4				
Redbro	57	37	7	0	0	80	60.43	-	> 0.9
Ross 308	53	47	0	0	0	80	60.57		
	**Week 7**								
	LS0	LS1	LS2	LS3	LS4				
Redbro	55	38	7	0	0	80			> 0.8^1^

^1^p value for a slaughter weight comparison between week 7 Redbro and week 6 Ross 308 broilers.

### Production data

**Fig 5:**
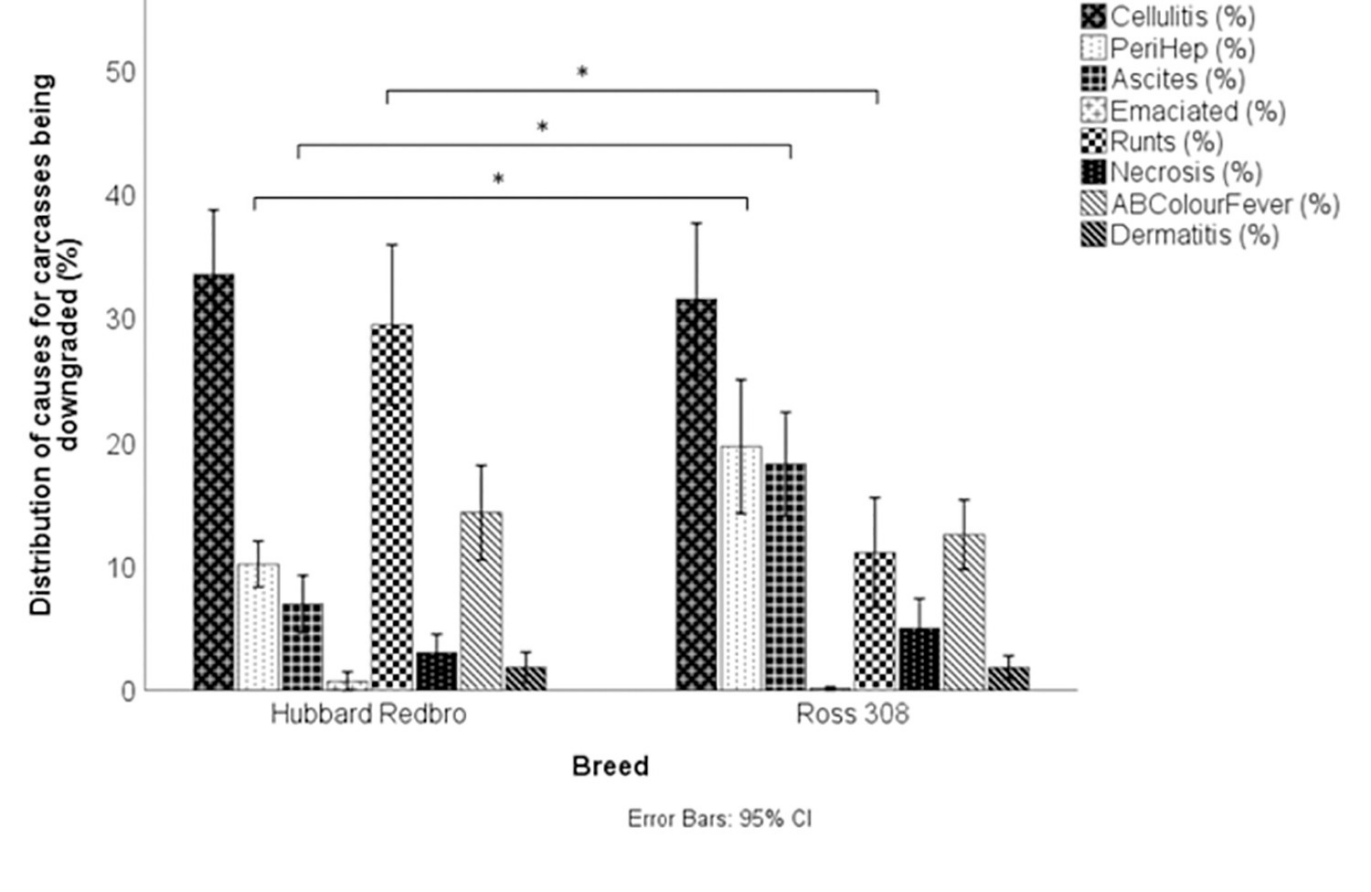
The distribution of causes of carcass downgrades for Redbro broilers and Ross 308 broilers at slaughter. * denotes a significant difference between breed in the % of broilers given that downgrade category.

Ross 308 broilers grew at an average of 65 g per day and had an average slaughter age of 38.7 days, reaching an average weight of 2.52 kg. Hubbard Redbro broilers grew at a slower 53 g per day, with an average slaughter age of 44.2 days at around 2.32 kg (Table 5). There was a significant effect of breed on the percentage of carcasses that were downgraded, total mortality, mortality (not including culls), and the overall percentage of culls (Table 5). The type of cull carried out was not affected by breed, with similar levels of leg culls, size culls and other (Table 5). There was also no effect of breed on average levels of hock burn or pododermatitis (Table 5). Of the carcasses downgraded at the abattoir, the distribution of the reasons for their downgrade are displayed in Fig 5. A larger percentage of the downgrades were attributed to perihepatitis (F(1,16) = 20.30. p < 0.001) and ascites (F(1,16) = 19.18, p < 0.001) in Ross 308s compared to Redbro, and a significantly larger percentage of Redbro broilers were scored as runts compared to Ross 308s (F(1,16) = 19.33, p < 0.001). When mortality and cull data was inspected by day there was a significant effect of breed on most days (Table 5). Significantly higher mortality (birds found dead in the house) was recorded in the Ross 308s compared to the Redbro on day 3 (F(1,16) = 14.73, p = 0.005), day 7 (F(1,16) = 20.44, p < 0.001), day 14 (F(1,16) = 25.90, p < 0.001), day 21 (F(1,16) = 47.17, p < 0.001) and day 28 (F(1,16) = 101.79, P < 0.001). Significantly more Ross 308s were culled on day 14 (F(1,16 = 24.60, p < 0.001) and day 28 (F(1,16) = 9.14, P = 0.040) compared to Redbro broilers.

**Table 5:** Slaughter data from 17 production cycles, comparing Ross 308 broilers with slower growing Redbro broilers. All percentage values represent the % of head placed (flock size at the beginning of the cycle). Raw mean values and standard deviations (±) presented.

	Breed		
	Redbro	Ross 308	p value^1^
Slaughter age	44.18 days ± 1.78	38.65 days ± 1.27	
Slaughter weight	2.32 kg ± 0.12	2.52 kg ± 0.14	
Average daily weight gain (g)	53	65	
Planned stocking density (kg/m^2^)	30	30	
Pre-thin stocking density (kg/m^2^)	29.46 ± 1.46	29.69 ± 1.52	
Clearing stocking density (kg/m^2^)	22.07 ± 3.16	24.66 ± 2.97	
Days between thin and clearing	6.94 ± 1.34	6.94 ± 0.66	
Downgrades (%)	0.67 ± 0.31	0.95 ± 0.58	0.040
Dead on arrival (%)	0.09 ± 0.03	0.10 ± 0.03	NS
Total Mortality (mort + culls; %)	2.18 ± 0.60	3.76 ± 1.45	< 0.001
Mortality (%)	1.43 ± 0.26	2.38 ± 0.64	< 0.001
Culls (%)	0.74 ± 0.47	1.39 ± 1.11	0.012
% of culls that were leg	50.11 ± 21.30	48.89 ± 16.71	NS
% of culls that were size	42.93 ± 19.18	42.38 ± 15.23	NS
% of culls that were other	6.96 ± 7.45	8.74 ± 10.14	NS
Average hockburn (%)	7.39 ± 5.02	7.17 ± 5.82	NS
Average pododermatitis (%)	29.19 ± 30.01	38.09 ± 31.47	NS
Day 3 mortality (%)	0.32 ± 0.12	0.66 ± 0.36	0.005
Day 7 mortality (%)	0.59 ± 0.18	1.11 ± 0.49	< 0.001
Day 14 mortality (%)	0.31 ± 0.09	0.45 ± 0.12	< 0.001
Day 21 mortality (%)	0.28 ± 0.08	0.40 ± 0.11	< 0.001
Day 28 mortality (%)	0.16 ± 0.03	0.29 ± 0.07	< 0.001
Day 3 culls (%)	0.08 ± 0.08	0.11 ± 0.10	NS
Day 7 culls (%)	0.21 ± 0.13	0.30 ± 0.21	NS
Day 14 culls (%)	0.16 ± 0.07	0.24 ± 0.13	< 0.001
Day 21 culls (%)	0.16 ± 0.20	0.26 ± 0.28	NS
Day 28 culls (%)	0.11 ± 0.12	0.22 ± 0.22	0.040

Significance set at p < 0.05. NS = non-significant.

## Discussion

The purpose of this study was to compare the health and welfare of a conventional fast growing broiler (Ross 308) and a slower growing breed (Hubbard Redbro) on commercial farms. We found that breed had a significant effect on a number of outcomes, with slower growing broilers demonstrating improved leg health measures, perch use, feather cover and levels of play behaviour. Slower growing broilers were more reactive to human observers during avoidance testing but displayed no difference in the way they reacted to novel objects compared to the fast growing breed. We saw no notable difference in the types of behaviours and level of resting performed by each breed during scan sampling, however Redbro broilers did have longer activity bouts in their final week than Ross 308s. No significant differences in feather cleanliness, litter condition or environmental measures were found, although all tended to be at good levels throughout the study. Production data revealed that Redbros took 5.5 days longer on average to reach the chosen slaughter weight they were cleared at by producers, which was lighter than the average weight reached by Ross 308s. Redbro’s had lower mortality levels and fewer culls across the cycle, and were less likely to have their carcasses downgraded at slaughter.

Although gait score worsened as broilers aged in this study, the number of lame broilers with a gait score of ≥ 3 was very low (~ 1% for both breeds in their final week). Recent studies have reported lameness in fast growing broiler flocks to be around 3% [20], 16% [7], 19% [21], 26–37% [8] and 30% [22]. This variation is probably due to some combination of the different breeds used, housing conditions (from pens to working farms), management style (level of leg culling), stocking density, age at assessment and the subjective nature of gait scoring. A high litter quality and low stocking density (30 kg/m^2^) may have contributed to the low gait scores seen in this study [23,24]. Despite the generally good gait scores, slow growing broilers still displayed significantly improved walking ability compared to fast growing broilers and took significantly longer to sit down during latency to lie tests. This indication of better leg health is consistent with a number of recent studies exploring the advantages of rearing slower growing breeds [7,8]. The difference in weight between the two breeds at each testing period may go some way to explain the results, with Redbro broilers being cleared at a lighter weight than Ross 308s. Body weight has been found to be associated with walking ability, with broilers demonstrating improved locomotion when 50% of their body weight is alleviated [25]. Other authors have found that when variations in live weight were factored into their study, they lost any difference in gait score between fast and slow growing broilers [26]. In the present study, Redbro broilers displayed lower gait scores than their fast growing counterparts once they approached slaughter weight, which may suggest an increase in resilience to a similar physical load. A reduction in skeletal issues has been thought to occur when growth rates are slowed down by giving the broiler’s skeleton more time to adapt to the increasing body weight. Evidence of improved bone mineralisation and bone quality measures have been found in slower growing breeds, which could equate to reductions in skeletal deformities, infections and breakages among a flock [27–30].

Contrary to our expectations, the apparently better leg health among slow growing broilers was not reflected in their activity levels. We found no significant difference in the types of behaviours observed during scan sampling, including the levels of sitting, resting and locomotion. Several studies have reported that slow growing broilers are more active than fast growing breeds [8,31,32]. The slower growing Redbro broilers assessed in this study had a higher daily weight gain and shorter rearing period than the birds used in these other studies. As activity levels are linked with body weight [25,32], it may be that the difference in weight between Redbro and Ross 308 broilers was not substantial enough to lead to significant differences in their behaviour. However, activity bouts observed in the Redbro broilers were significantly longer than those in the Ross 308s when both breeds reached their respective final weights. We also observed markedly higher perch use in the slower growing Redbro broilers, with over four times as many Redbro broilers observed on the perches compared to Ross 308s. This is consistent with a number of studies reporting increased levels of perching in slower growing broilers [8,32,33]. Redbro perch use increased to a peak in week 5 before reducing in their final weeks while perch use remained low throughout the cycle in Ross 308s, although the length of both breeds perching bouts was similar. Once both breeds reached their final weights, perch occupancy was still significantly higher among the slower growing broilers. Redbros also tended to be more successful at jumping onto the perches than Ross 308s, and made significantly more attempts. Although there was no clear difference in their general behaviours during scan samples, this increase in perch use and longer activity bouts at slaughter weight does suggest the slower growing breed may be more resilient to a high body weight and more physically capable of interacting with the enrichments provided.

As well as displaying better leg health measures, slow growing broilers withdrew from an approaching observer significantly earlier than fast growing broilers. This higher level of reactivity typically suggests they are more fearful. However, there has been some debate about the suitability of avoidance tests as a measure of fearfulness in broilers. Fast growing broilers have been reported to be less likely to approach an observer during a touch test compared to a slow growing breed [7]. However, the study’s authors acknowledge that these tests are often confounded by fast-growing broilers being less physically able or less motivated to approach an observer. Vasdal et al. [34] found that higher gait scores in broilers were associated with reduced withdrawal from humans, creating a likely false indication of less fearful animals. Using an alternative approach-based test to measure avoidance, we similarly found that Ross 308 broilers had shorter avoidance distances than Redbros. However, we found no difference in the way each breed approached or interacted with novel objects, an additional test of fearfulness in poultry [35]. Given the significant difference in gait scores and latency to lie tests, it is likely that the approach test provides a further measure of the fast-growing broilers reduced locomotor ability. Withdrawal distances also reduced with age, which may be a function of reduced locomotion or reduced fearfulness. All broilers took less time to approach novel objects and were more likely to interact with them as they aged in this study, which is consistent with a lower avoidance of novel objects seen over time in laying hens [36]. The majority of fear related tests are only performed on broilers at a single time point, however our result is contrary to previous similar studies indicating that interaction with novel objects decreased as birds aged [16,37].

Play is typically associated with positive welfare, positive emotional states and the absence of stressors [38]. This area of research for poultry is fairly new, and the association between play behaviour and broiler welfare is not yet clear [39]. Enriching the environment of fast-growing broilers has been shown to have a negligible [19,34] or negative effect [40] on the display of varying lists of play behaviours. However, Rayner et al. [7] found that breed was a significant predictor of play, with the least play seen in a fast growing breed compared to two slower growing broiler strains. We similarly observed significantly higher levels of frolicking and sparring in Redbro broilers compared to the Ross 308s, with play reducing to a similar level for both when they reached a similar final weight. It is likely that better overall leg condition and a lighter body weight among Redbro broilers made them more physically capable of displaying active play behaviours throughout the cycle [5,41]. It is also possible that physical limitations, reduced use of enrichments and a poorer walking ability induced a more negative mental state among Ross 308 flocks, reducing the expression of play [38]. The effect of age on observations of play behaviour was consistent with similar studies of stimulated play [7,19]. Play was initially low, increasing to peak in week 5 before reducing until the birds reached slaughter weight, in line with their declining physical ability [4]. Studies exploring play in undisturbed rather than disturbed areas find that less play is performed overall, but that the highest level is seen in younger birds before it declines linearly as birds age [7,34,40]. Using a walk-through is a successful method of stimulating play in an observable area and generating space for broilers to express a variety of behaviours. This effect becomes more pronounced as the other available space becomes restricted for older birds, which is likely to be why we see an initial increase in the middle of the cycle. It would be interesting to further explore the difference in how play is expressed in disturbed and undisturbed areas.

Feather cover is increasingly being factored into breeding programmes as a welfare goal [42]. Feathers are unique structures that play an integral role in the life of all birds. For broilers, this role is largely limited to providing physical protection from scratches and a physical barrier between skin and wet litter. Although feathers typically play a role in maintaining body temperature, automatically controlled heating systems in broiler housing have been designed to prevent broilers losing feed energy through thermoregulation [43]. However, broilers still devote substantial amounts of time to comfort behaviours associated with their feathers, including preening and dustbathing, which suggests feathers may play a more fundamental role in their well-being. From week 4 of the production cycle onwards, we found that Redbro broilers had significantly better feather cover than Ross 308s, including once both breeds reached their final weights. Dixon [8] similarly found that a slower growing broiler strain had better feather cover than three faster growing breeds. We saw an initial worsening of feather cover in both breeds in week 4, probably due to patchiness caused by the replacement of down with adult feathers. Redbro broilers appeared to develop their full feathering more rapidly, with the clearest difference in scores between the two breeds seen in week 5 of the production cycle. Dirty feathers were rare in this study, and no significant difference was noted between the two breeds. Dixon [8] found that slow growing broilers had better feather cleanliness compared to three fast growing breeds, as well as lower levels of hockburn, and suggested that their higher activity levels may have reduced any contact with the litter. We saw no substantial difference in activity levels between Redbro and Ross 308 broilers, and all houses had generally good quality litter. This may account for the overall high feather cleanliness and the similar levels of contact dermatitis in both breeds. There was only a temporary difference in litter condition between the two breeds, with worse litter scores in the Ross 308 houses for week 4 only. Previous studies have reported litter quality being lower in fast growing compared to slow growing houses [7]. It is possible that there was an effect of breed on litter, but that this was masked by the maintenance woodshavings that farmers distributed to maintain dry bedding. Regardless, farmers were able to maintain both houses to a good standard, which is likely to have a significant effect on overall welfare parameters [44]. Ammonia levels increased over the production cycle, as expected, but there was no difference between the two breeds for ammonia concentration or dust levels.

There were significant differences found in the mortality, health and carcass quality of the two breeds. Slower growing Redbro broilers recorded lower mortality, fewer culls and fewer carcasses downgraded at the abattoir. There was also a difference in the type of downgrades seen at the abattoir, with higher levels of perihepatitis, higher levels of ascites and a lower frequency of runts seen in Ross 308s. Consistent with recent research [7,8], these results suggest that the slower growing strain was generally healthier throughout the study. A number of their outcomes also compare favourably to other slow growing strains, despite having a generally faster growth rate. For example, a slow growing strain studied by Rayner et al. [7] under similar commercial conditions displayed similar levels of mortality and carcass downgrades, but were slaughtered an average of a day later at around 20 g lighter (47 g/day). A slow growing strain in Dixon [8] also had a slower rate of growth (46 g/day) compared to Redbro broilers but reported similar gait scores at slaughter weight. There is currently no clear definition of a slow growing broiler, with slow growth considered to be 26 g/day by Label Rouge [45], < 50 g/day by Global Animal Partnership [46] and 48–50 g/day by the Federal Office for Agriculture and Food [47]. Growth rates and production outcomes can have a large impact on the economics of farms and the practicalities of adopting a slower growing breed on a large scale. Identifying strains of broiler that demonstrate improved welfare outcomes while also maintaining productivity is therefore likely to lead to greater uptake by the poultry industry. Assessing these strains under commercial conditions will also produce the most relevant evidence base for the industry.

## Conclusions

We found that slower growing Hubbard Redbro broilers demonstrated a number of better health and welfare outcomes when compared to conventional Ross 308 broilers under commercial conditions. Although there were only minor differences noted in their general behaviour, better leg health and feathering was observed among the slower growing flocks. Redbro broilers also appeared to be more physically able to make use of the perches available, move into cleared areas of the house to play, and react to approaching observers. Redbro broilers were cleared an average of 5.5 days later than the fast growing breed at a lighter weight, but they appeared to be healthier throughout the cycle. Lower levels of mortality, fewer culls and a lower number of carcass downgrades were recorded among Redbro flocks. Environmental conditions have been shown to have a large impact on the health and welfare of intensively reared broilers. With no set definition of slow growth, on farm studies, where possible, will provide the industry with the most relevant advice on which slower growing strains demonstrate better welfare outcomes compared to fast growing broilers.
